# Restoring Ventricular Geometry: Left Ventricular Reconstruction in a Patient with a Giant Left Ventricular Aneurysm and End-Stage Heart Failure

**DOI:** 10.3390/jcm15155937

**Published:** 2026-07-30

**Authors:** Moldovan Horatiu, Dobra Irina, Robu Mircea, Safta Maria Sabina, Andrada Guta, Voicu Alexandra, Gabriel Goretzki, Lucian Dorobantu, Menicanti Lorenzo, Ondin Zaharia

**Affiliations:** 1 Faculty of Medicine, “Carol Davila” University of Medicine and Pharmacy, 050474 Bucharest, Romania; h_moldovan@hotmail.com (M.H.); irina.dobra@drd.umfcd.ro (D.I.); andrada.c.guta@gmail.com (A.G.); alexandra.voicu@icloud.com (V.A.); ondin.zaharia@gmail.com (O.Z.); 2 Academy of Romanian Scientists, 050711 Bucharest, Romania; 3 Department of Cardiovascular Surgery, Clinical Emergency Hospital Bucharest, 014461 Bucharest, Romania; 4 Department of Anesthesia and Intensive Care, Faculty of Medicine, Titu Maiorescu University, 040051 Bucharest, Romania; 5 Faculty of Medicine, Titu Maiorescu University, 040441 Bucharest, Romania; 6 Policlinico San Donato, 20097 Milan, Italy; lorenzo.menicanti@grupposandonato.it

**Keywords:** post-infarction left ventricular aneurysm, surgical ventricular reconstruction, endoventricular circular restoration, heart failure, cardiac magnetic resonance, intra-aortic balloon pump, coronary artery bypass grafting

## Abstract

Post-infarction left ventricular aneurysm is an uncommon but severe mechanical complication of transmural myocardial infarction, particularly in patients with delayed presentation or incomplete myocardial salvage. It may lead to profound distortion of left ventricular geometry, adverse remodelling, intraventricular thrombosis, mitral regurgitation, pulmonary hypertension, and advanced heart failure. We report the case of a 65-year-old male patient referred two months after a late-presenting anterior ST-segment elevation myocardial infarction caused by proximal occlusion of the left anterior descending coronary artery. At admission, the patient presented with severe decompensated heart failure, low-output status, multiorgan dysfunction, and a left ventricular ejection fraction of 12%. Transthoracic echocardiography and cardiac magnetic resonance imaging demonstrated a giant apical left ventricular aneurysm involving approximately 75% of the ventricular cavity, partial intraluminal thrombosis, extensive transmural scarring in the left anterior descending territory, and imaging features suggestive of a chronic contained free-wall rupture/pseudoaneurysmal component. Following multidisciplinary evaluation, the patient underwent surgical ventricular reconstruction using an endoventricular circular restoration technique guided by an intraventricular balloon sizer, combined with left internal thoracic artery bypass grafting to the left anterior descending artery. The early postoperative course required temporary inotropic, vasopressor, inhaled nitric oxide, and intra-aortic balloon pump support, followed by progressive haemodynamic recovery. The patient was discharged on postoperative day seven with functional improvement to NYHA class II. At six-month follow-up, he remained clinically stable without overt signs of heart failure, and echocardiography showed preserved ventricular geometry and improvement of left ventricular ejection fraction to 45%. This case highlights the potential role of carefully planned, balloon-guided surgical ventricular reconstruction in selected patients with giant post-infarction left ventricular aneurysms and end-stage heart failure when residual viable myocardium is present.

## 1. Introduction

Heart failure (HF) remains a major global health burden, with ischemic heart disease representing one of its leading etiologies [1].

A post-infarction left ventricular aneurysm is a severe mechanical complication of transmural myocardial infarction, historically reported in up to 30–35% of untreated cases in the pre-reperfusion era [2]. It occurs predominantly in patients with large infarcts, delayed presentation, or absence of timely myocardial revascularization according to contemporary STEMI guideline recommendations [3]. Structurally, it consists of a thinned, fibrotic, non-contractile or dyskinetic ventricular segment that distorts left ventricular geometry, increases wall stress, and promotes adverse remodeling, thereby contributing to progressive systolic dysfunction and HF. 

Surgical ventricular reconstruction (SVR) is designed to exclude or resect the akinetic or dyskinetic scarred myocardial segment and to restore a more physiological left ventricular (LV) geometry. The role of SVR, however, remains debated. In the STICH trial, the addition of SVR to CABG produced a greater reduction in LV end-systolic volume compared with CABG alone, but did not translate into a significant reduction in all-cause mortality or cardiac hospitalization during follow-up [4]. Nevertheless, subsequent analyses and contemporary surgical series have suggested that the neutral findings of STICH may not be generalizable to all patients, particularly because the benefit of SVR appears highly dependent on appropriate patient selection, the presence of a clearly defined scarred or aneurysmal segment, the degree of postoperative LV volume reduction, and the achievement of complete myocardial revascularization [5,6,7]. Thus, although SVR should not be considered a routine adjunct to CABG in all patients with ischemic LV dysfunction, it remains a relevant therapeutic option for carefully selected patients with severe ischemic remodeling, discrete scar-related dyskinesia or aneurysm, and surgically amenable coronary artery disease.

Here we present a case of a 65-year-old male with late-presentation anterior STEMI, resulting in a gigantic LV aneurysm encompassing 75% of the LV cavity, and an EF of 12%, who underwent successful SVR combined with CABG.

## 2. Case Presentation

A 65-year-old male patient was referred to our Cardiovascular Surgery Department because of progressive signs and symptoms of advanced heart failure, including dyspnoea at rest, peripheral oedema, severely reduced exercise tolerance, and clinical evidence of low cardiac output. The patient was 178 cm tall, weighed 82 kg, with a calculated body surface area (Mosteller formula) of 2.01 m^2^.

His medical history was notable for a late-presenting anterior ST-segment elevation myocardial infarction (STEMI) that had occurred two months earlier, with the patient seeking medical attention approximately one week after symptom onset. At that time, coronary angiography demonstrated proximal occlusion of the left anterior descending (LAD) coronary artery, and the patient was managed conservatively. During the subsequent course, he experienced episodes of sustained ventricular tachycardia, for which an implantable cardioverter-defibrillator (ICD) was implanted. At admission to our institution, his haemodynamic status was severely compromised, with arterial blood pressure of 90/40 mmHg, regular heart rhythm at 65 beats per minute, oliguria progressing to oligoanuria, and peripheral oxygen saturation of 93% while breathing room air.

**Figure 1:**
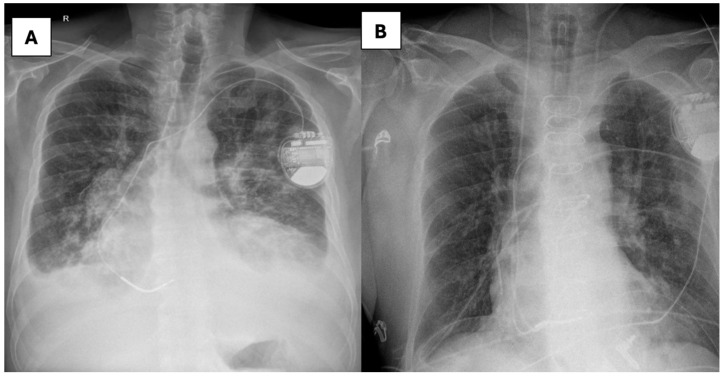
Chest radiographs obtained before and after surgical ventricular reconstruction. (**A**) Preoperative chest radiograph demonstrating marked cardiomegaly, bilateral pleural effusions, and pulmonary vascular/interstitial congestion consistent with advanced congestive heart failure. An implantable cardioverter-defibrillator (ICD) is visible in the left pectoral region. (**B**) Early postoperative chest radiograph showing a reduction in cardiac silhouette size and improvement of pulmonary congestion following surgical treatment.

The electrocardiogram showed sinus rhythm with frequent ventricular premature beats and persistent Q waves with residual ST-T abnormalities in the anterior leads, consistent with a previous extensive anterior myocardial infarction. Preoperative chest radiography (Figure 1A) revealed severe cardiomegaly with marked enlargement of the left cardiac silhouette, reflecting extensive adverse left ventricular remodeling secondary to a giant post-infarction anterior/apical aneurysm. Bilateral pleural effusions, greater on the left, and diffuse pulmonary vascular/interstitial congestion with bibasal alveolar opacities were present, consistent with decompensated congestive heart failure. A permanent left-sided pacemaker was also visible.

Laboratory tests demonstrated systemic hypoperfusion and multiorgan dysfunction, including elevated serum lactate of 4 mmol/L, anaemia with haemoglobin of 8.7 g/dL, renal dysfunction with serum creatinine of 2.3 mg/dL and urea of 125 mg/dL, as well as hepatic injury consistent with congestive hepatopathy and hypoperfusion, with AST/ALT values of 215/128 IU/L.

**Figure 2:**
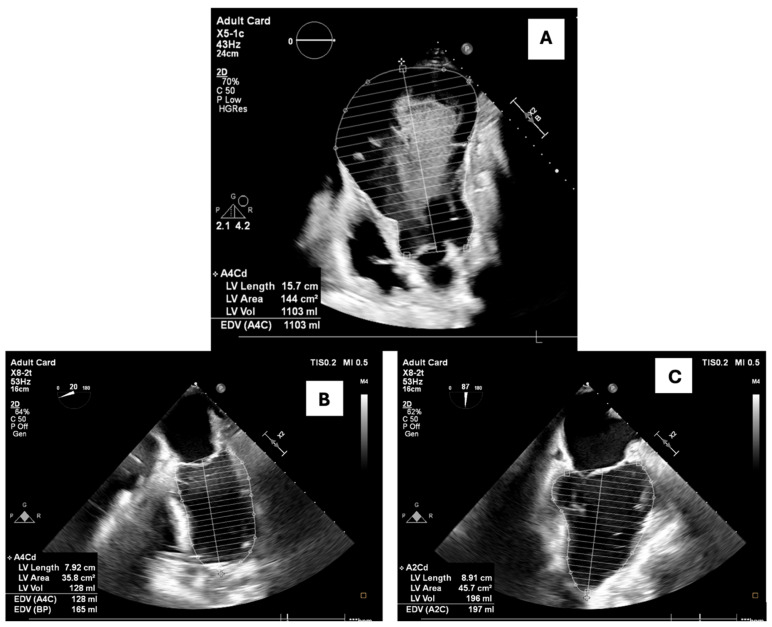
Preoperative and postoperative transthoracic echocardiography. (**A**) Apical four-chamber view demonstrating a giant post-infarction left ventricular aneurysm associated with massive ventricular dilatation (LVEDV 1103 mL). (**B**,**C**) Early postoperative apical four-chamber and two-chamber views showing successful ventricular restoration with recovery of a more physiological left ventricular cavity shape and a substantial reduction in LV end-diastolic volume (LVEDV 165 mL, biplane Simpson method).

Transthoracic echocardiography revealed demonstrated extreme left ventricular dilatation secondary to a giant anterior-apical aneurysm, resulting in severe distortion of ventricular geometry and near-spherical remodeling. The aneurysmal cavity occupied the majority of the left ventricular chamber, with extensive thinning and akinesia/dyskinesia of the affected segments (apical four-chamber view) (Figure 2A). Quantitative measurements obtained in the apical four-chamber view revealed a left ventricular longitudinal dimension of 15.7 cm, a calculated left ventricular cavity area of 144 cm^2^. Quantitative measurements were obtained by two-dimensional transthoracic echocardiography using the biplane Simpson method. Despite the marked distortion of left ventricular geometry caused by the giant aneurysm, the calculated left ventricular end-diastolic volume (LVEDV) was 1103 mL, reflecting extreme ventricular dilatation.

The left ventricular ejection fraction was severely depressed at 12%, consistent with end-stage ischemic cardiomyopathy. The right ventricle was also dilated, with a basal diameter of 45 mm, and showed moderate systolic dysfunction, as reflected by reduced tricuspid annular plane systolic excursion (TAPSE 13 mm), decreased tissue Doppler systolic velocity of the tricuspid annulus (S′ 8 cm/s), and reduced fractional area change. Moderate functional mitral regurgitation was present, secondary to severe left ventricular remodelling and leaflet tethering. The estimated right ventricular–right atrial systolic gradient was 50 mmHg, suggesting moderate pulmonary hypertension. No pericardial effusion was detected.

**Figure 3:**
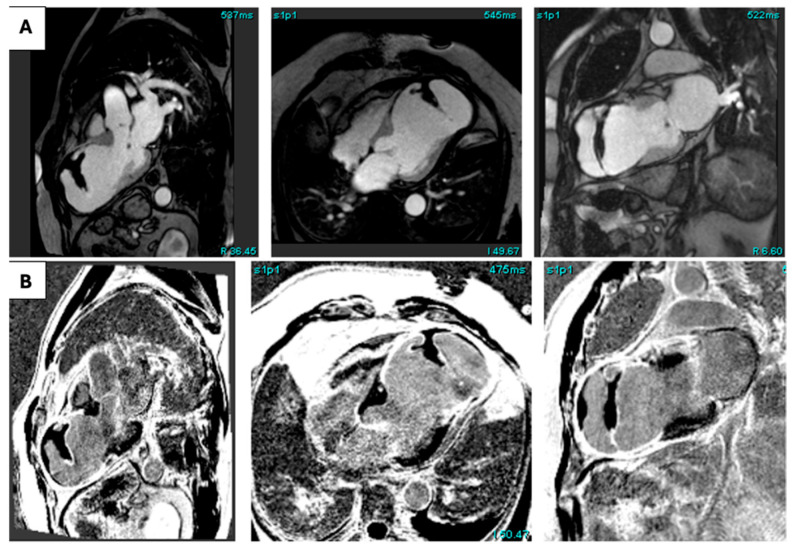
Preoperative cardiac magnetic resonance imaging (CMR). (**A**) Cine images demonstrating a giant post-infarction left ventricular aneurysm involving the apical and anterior segments, associated with severe left ventricular dilatation and adverse remodelling. (**B**) Late gadolinium enhancement sequences showing extensive transmural scar formation of the aneurysmal wall and partially organized mural thrombus. A discontinuity within the aneurysmal wall with a thin overlying tissue layer is visible, raising suspicion for a chronic contained free-wall rupture.

The findings were confirmed and further characterised by cardiac magnetic resonance imaging (CMR) (Figure 3A,B). CMR demonstrated a giant left ventricular aneurysm involving the anterior, apical, and anterolateral segments, resulting in severe distortion of left ventricular geometry and marked chamber dilatation. The aneurysmal cavity occupied a substantial proportion of the left ventricular volume and exhibited a thin dyskinetic wall with near-complete loss of contractile function. A large mural thrombus was identified along the aneurysmal wall, predominantly at the apical region. Global left ventricular systolic function was severely depressed, with extensive adverse ventricular remodeling. Late gadolinium enhancement sequences revealed transmural fibrosis and scar formation involving the aneurysmal segments, consistent with chronic extensive anterior myocardial infarction. Areas of marked wall thinning and discontinuity of the infarcted myocardium were associated with a contained outpouching, raising suspicion for a chronic contained free-wall rupture (pseudoaneurysmal component) within the giant aneurysm. No viable myocardium was identified within the scarred aneurysmal region. The examination allowed accurate delineation of the scarred and viable myocardial segments, assessment of thrombotic burden within the aneurysmal cavity, and detailed preoperative evaluation of left ventricular geometry for surgical ventricular reconstruction planning. Given the patient’s critical clinical condition, the absence of a suitable donor heart precluded cardiac transplantation as an immediate therapeutic option. Following multidisciplinary team discussion and thorough preoperative planning, SVR was selected as the most appropriate surgical intervention. Preoperative assessment included coronary angiography, which confirmed proximal LAD occlusion amenable to bypass, complete echocardiographic and CMR assessment of ventricular geometry. Preoperatively, the patient received intravenous loop-diuretic therapy with furosemide administered by continuous infusion at 5 mg/h, with subsequent dose adjustment according to urine output, fluid balance, renal function, and clinical signs of congestion. Oral beta-blocker therapy with metoprolol 50 mg daily was continued as hemodynamically tolerated. Therapeutic anticoagulation was with intravenous unfractionated heparin, initiated with a 600 IU continuous infusion, with dose titration to maintain an activated partial thromboplastin time (aPTT) between 50 and 60 s. Because of the initial hypotension and systemic hypoperfusion, renin–angiotensin system inhibition was withheld during the unstable phase. 

**Figure 4:**
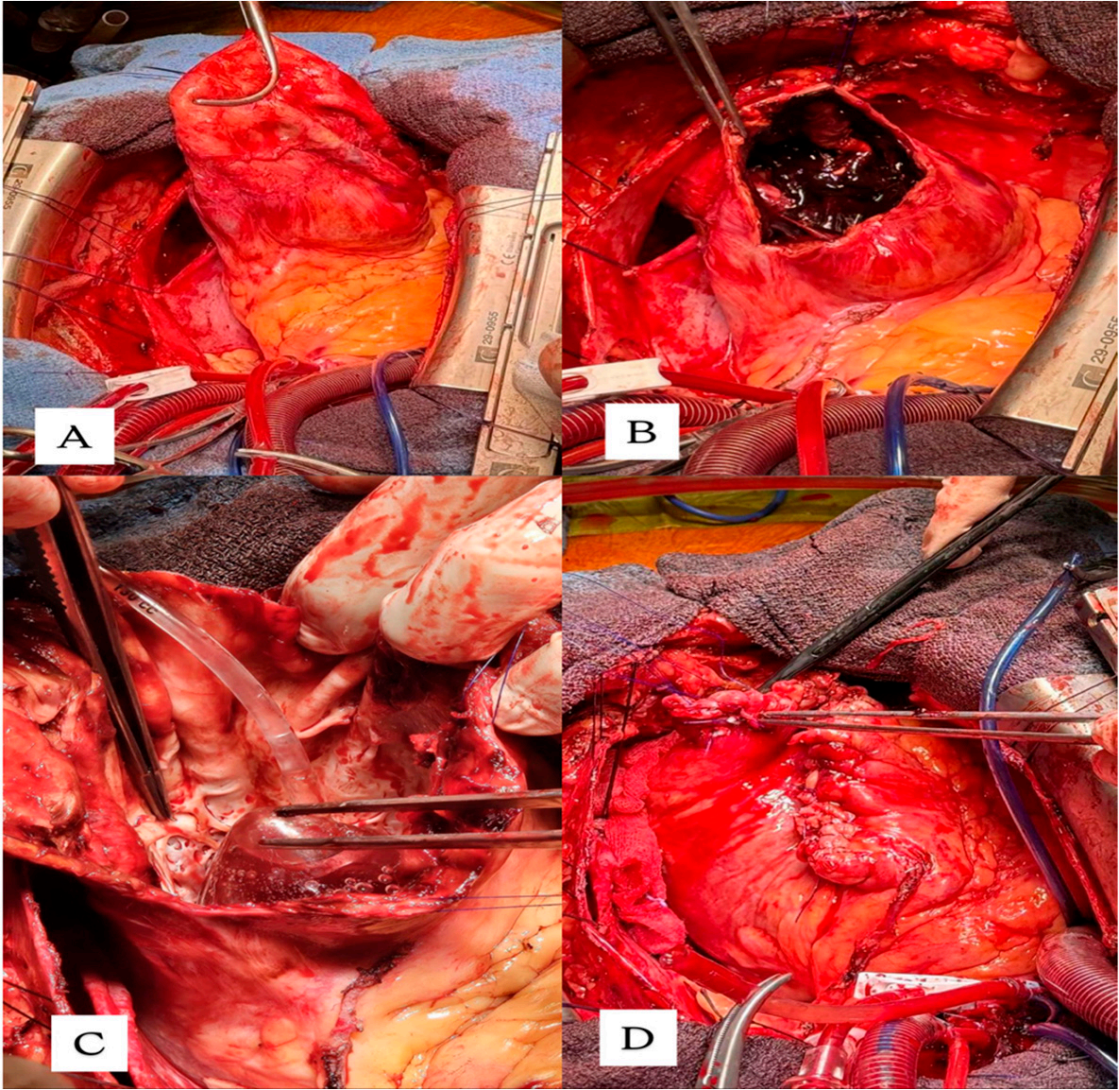
Intraoperative images illustrating surgical ventricular reconstruction. (**A**) Giant left ventricular apical aneurysm exposed through median sternotomy, demonstrating severe ventricular enlargement and distortion of normal cardiac geometry. (**B**) Opening of the aneurysmal sac, revealing a large aneurysmal cavity containing organized mural thrombotic material. (**C**) Endoventricular inspection after thrombectomy, showing the transition zone between viable myocardium and fibrotic scar tissue. A sizing balloon is positioned within the residual left ventricular cavity to guide ventricular reconstruction and restoration of an appropriate ventricular volume and geometry. (**D**) Completion of surgical ventricular reconstruction using endoventricular circular purse-string plication of the scar–viable myocardium interface followed by aneurysm exclusion and linear closure, restoring a more physiological left ventricular configuration.

After median sternotomy and institution of cardiopulmonary bypass, the heart was arrested using antegrade cold blood cardioplegia. Following cardioplegic arrest, the giant left ventricular aneurysm was carefully dissected from dense pleuro-pericardial adhesions, allowing complete exposure of the aneurysmal wall and safe identification of the surrounding viable myocardium (Figure 4). The aneurysmal sac was then opened at its most prominent and thinned portion, and a large amount of organized thrombotic material was removed from within the aneurysmal cavity. The left ventricular cavity was thoroughly inspected, and the transition zone between the fibrotic, scarred aneurysmal myocardium and the residual viable contractile myocardium was clearly identified.

Surgical ventricular reconstruction was performed according to the principles of endoventricular circular restoration. A circumferential purse-string suture using 3-0 Prolene was placed at the level of the border zone between scarred and viable myocardium, thereby excluding the non-contractile aneurysmal portion from the functional left ventricular cavity. Before tightening the suture, a saline-filled intraventricular balloon sizer was introduced into the residual left ventricular cavity to guide reconstruction, prevent excessive volume reduction, and restore a more physiological elliptical ventricular geometry. In accordance with commonly described surgical ventricular restoration techniques, the target balloon volume was selected according to body surface area, with most authors recommending approximately 50–60 mL/m^2^ to optimize the final residual ventricular volume. 

The patient’s calculated body surface area was 2.01 m^2^. Based on current surgical ventricular restoration practice (50–60 mL/m^2^), the target balloon volume was approximately 100–120 mL; therefore, a 100 mL balloon was selected to avoid both residual ventricular over-dilatation and over-reduction in the left ventricular cavity. This sizing strategy is consistent with the concept introduced in contemporary SVR techniques, where an intraventricular balloon or mannequin is used to standardize the final ventricular volume and preserve the long-axis orientation of the reconstructed ventricle. 

Once the optimal ventricular geometry was obtained, the purse-string suture was progressively tightened over the balloon, reducing the aneurysmal orifice and recreating the functional left ventricular cavity at the level of viable myocardium. The balloon was subsequently deflated and removed. The remaining aneurysmal wall was then closed over the reconstruction with a haemostatic suture line, using the aneurysmal “jacket” as an external reinforcing layer to ensure haemostasis and to provide additional support to the reconstructed ventricle.

Last but not least, myocardial revascularisation was completed via aorto-coronary bypass using the left internal thoracic artery anastomosed to the LAD II ensuring adequate perfusion to the residual viable myocardium. 

The immediate postoperative course was initially marked by haemodynamic instability related to severely impaired preoperative ventricular function and residual right ventricular dysfunction. At weaning from cardiopulmonary bypass, pharmacological circulatory support was required, consisting of positive inotropic therapy with dobutamine and moderate-dose norepinephrine for vasopressor support. Inhaled nitric oxide was administered because of moderate right ventricular dysfunction and pulmonary hypertension, and an intra-aortic balloon pump was inserted to provide additional mechanical circulatory support and reduce left ventricular afterload.

Intraoperative transoesophageal echocardiography, using the biplane Simpson method, demonstrated successful exclusion of the aneurysmal cavity with a residual LVEDV of 165 mL (Figure 2B,C) and restoration of a more physiological left ventricular geometry, with an estimated left ventricular ejection fraction of 35–45%. Moderate mitral regurgitation persisted, while the right ventricle remained moderately dysfunctional, associated with moderate tricuspid regurgitation. No residual aneurysmal cavity or pericardial collection was identified.

The patient was transferred to the intensive care unit, where progressive haemodynamic improvement was observed. Inotropic and vasopressor support were successfully weaned by the second postoperative day. The intra-aortic balloon pump was removed on the third postoperative day, and the patient was subsequently transferred to the surgical ward. The total intensive care unit stay was three days. The remainder of the postoperative course was favourable, with gradual improvement in signs and symptoms of heart failure. The patient was discharged on the seventh postoperative day in stable clinical condition, with functional improvement to NYHA class II. Postoperative chest XR revealed a reduction in cardiac silhouette size and improvement of pulmonary congestion following surgical treatment (Figure 1B).

At discharge, the patient was prescribed optimised guideline-directed medical therapy for heart failure with reduced ejection fraction, including renin–angiotensin–aldosterone system inhibition with an angiotensin-converting enzyme inhibitor, beta-blockade, mineralocorticoid receptor antagonism, sodium–glucose cotransporter 2 inhibition, loop diuretic therapy as clinically indicated, and anticoagulation. Outpatient cardiology and cardiac surgery follow-up were arranged. Transthoracic echocardiography performed before discharge demonstrated persistent akinesia of the apical two-thirds of the anterior interventricular septum and anterior wall, as well as the apical one-third of the inferior septum and inferior wall, consistent with the pre-existing extensive anterior myocardial infarction. Left ventricular systolic function remained moderately to severely impaired, with an estimated left ventricular ejection fraction (LVEF) of 25–30% and a left ventricular outflow tract velocity–time integral (LVOT VTI) of 13 cm. Mild residual mitral regurgitation was present. Right ventricular function was preserved, with a basal right ventricular diameter of 36 mm and a tricuspid annular plane systolic excursion (TAPSE) of 17 mm. Pacing leads from the previously implanted cardioverter-defibrillator were visualized within the right heart chambers. The inferior vena cava measured 16 mm in diameter and showed no features suggestive of elevated right atrial pressure. No pericardial effusion was detected. A small hyperechoic structure measuring approximately 1 cm was identified at the left ventricular apex, most likely corresponding to postoperative suture material or adjacent pericardial fat tissue.

At six-month follow-up, the patient remained clinically stable, without overt signs or symptoms of congestive heart failure. Transthoracic echocardiography confirmed preserved postoperative ventricular geometry and further improvement in left ventricular systolic function, with an estimated left ventricular ejection fraction of 45%.

## 3. Discussion

This case illustrates a complex and severe mechanical complication of a late-presenting anterior myocardial infarction, characterised by the development of a giant left ventricular aneurysm with extensive adverse ventricular remodelling, partial intraluminal thrombosis, and imaging features suggestive of a chronic contained free-wall rupture/pseudoaneurysmal component. Although the incidence of large post-infarction left ventricular aneurysms has declined substantially in the contemporary era of early reperfusion therapy, they remain clinically relevant in patients with delayed presentation, incomplete myocardial salvage, or persistent proximal LAD occlusion [8,9]. In such cases, transmural myocardial necrosis may lead to progressive thinning and dyskinetic expansion of the infarcted segment, resulting in severe distortion of left ventricular geometry, increased wall stress, reduced contractile efficiency, functional mitral regurgitation, pulmonary hypertension, and ultimately advanced heart failure [8,10].

An important diagnostic consideration in the present case was the distinction between a true left ventricular aneurysm, a pseudoaneurysm, and a contained free-wall rupture. True aneurysms are characterized by a broad neck and a wall composed of thinned but continuous scarred myocardium, whereas pseudoaneurysms result from complete myocardial rupture contained by the pericardium and typically exhibit a narrow neck with absence of myocardial tissue in the aneurysmal wall. Contained free-wall rupture represents an intermediate entity in which myocardial discontinuity is sealed by adherent pericardium or organized thrombus. In our patient, cardiac magnetic resonance imaging demonstrated extensive transmural fibrosis involving the aneurysmal wall, confirming the diagnosis of a giant true aneurysm, while a focal area of myocardial discontinuity covered by a thin outer tissue layer suggested a chronic contained free-wall rupture rather than a pure pseudoaneurysm. This distinction was clinically relevant because it explained the coexistence of chronic ventricular remodeling with imaging features indicating an increased risk of mechanical complications.

The clinical presentation of our patient reflected the haemodynamic consequences of this extreme form of post-infarction remodelling. At admission, the patient had severe left ventricular systolic dysfunction, signs of low cardiac output and systemic hypoperfusion, renal and hepatic dysfunction, pulmonary congestion, and moderate right ventricular impairment. These findings emphasise that giant left ventricular aneurysms should not be regarded solely as anatomical abnormalities, but rather as dynamic contributors to progressive circulatory failure. The aneurysmal segment acts as a non-contractile or dyskinetic chamber that increases end-diastolic volume and wall tension while reducing the efficiency of systolic ejection. In addition, distortion of papillary muscle geometry and annular dilatation may contribute to functional mitral regurgitation, further aggravating volume overload and pulmonary hypertension [8,10,11].

The present case also highlights the challenges associated with the management of patients presenting late after a completed myocardial infarction. The patient was initially treated at a referring institution following delayed presentation with a chronic proximal LAD occlusion and subsequently developed progressive heart failure and sustained ventricular tachycardia, for which an implantable cardioverter-defibrillator was implanted for secondary prevention of sudden cardiac death. Because the patient was referred to our center only after the development of a giant left ventricular aneurysm and end-stage heart failure, decisions regarding coronary angiography and revascularization during the early post-infarction period had been made before referral. At the time of our evaluation, the therapeutic focus shifted toward definitive surgical management, combining left internal thoracic artery bypass grafting with surgical ventricular reconstruction to restore ventricular geometry and improve global cardiac function.

In this context, surgical ventricular reconstruction was selected as a therapeutic strategy aimed not only at excluding the aneurysmal and non-viable myocardium, but also at restoring a more physiological left ventricular shape and volume. The rationale of surgical ventricular reconstruction is based on the principle that ventricular geometry is a major determinant of myocardial performance. By reducing excessive ventricular volume, excluding scarred dyskinetic segments, and recreating a more ellipsoidal ventricular cavity, surgical reconstruction can decrease wall stress, improve mechanical efficiency, and enhance the contribution of the remaining viable myocardium [10,12].

Interpretation of ventricular volume measurements in patients with giant post-infarction aneurysms requires caution because severe distortion of left ventricular geometry challenges the assumptions underlying two-dimensional echocardiographic volumetric analysis. Preoperative measurements were obtained by transthoracic echocardiography using the biplane Simpson method, whereas the immediate postoperative assessment was performed intraoperatively using transoesophageal echocardiography. Although these techniques may yield different absolute volume estimates, both examinations consistently demonstrated profound preoperative ventricular dilatation and a marked reduction in left ventricular volume following surgical reconstruction. Cardiac magnetic resonance imaging served primarily to characterize myocardial scar, thrombus burden, and ventricular anatomy rather than for serial volumetric comparison.

Multimodal cardiovascular imaging was central to individualized decision-making in this case. Transthoracic echocardiography quantified the severity of biventricular dysfunction, valvular involvement, pulmonary hypertension, and extreme ventricular dilatation, whereas cardiac magnetic resonance provided complementary characterization of scar distribution, myocardial viability, mural thrombus, and the suspected contained free-wall rupture. Beyond diagnosis, the complementary use of these modalities supported risk stratification, assessment of surgical feasibility, and individualized planning of ventricular reconstruction. This approach is consistent with the broader role of cardiovascular imaging in early disease characterization, risk assessment, and personalized therapeutic decision-making [10]. 

Several surgical techniques have been described for the treatment of post-infarction left ventricular aneurysms, broadly classified into classical linear repair and endoventricular reconstruction [10,13]. Classical linear repair, as originally described by Cooley, consists of aneurysmectomy followed by direct linear closure, but may not adequately restore physiological left ventricular geometry, particularly in large anterior aneurysms [10,13,14]. In contrast, endoventricular reconstruction, popularized by Dor, excludes the scarred myocardium from within the ventricular cavity using a circumferential suture placed at the border between viable and non-viable myocardium, with or without patch implantation depending on aneurysm morphology and the desired residual ventricular volume [12,13,15]. This reconstructive approach aims to restore a more physiological ventricular shape while preserving an adequate functional cavity [10,13].

The balloon-guided reconstruction used in the present case follows the principles of endoventricular circular restoration. The use of an intraventricular balloon or mannequin sizer was subsequently refined and popularised by Menicanti as a method to objectively define the final left ventricular cavity. The balloon is usually filled according to body surface area, most commonly to approximately 50–60 mL/m^2^, in order to optimise the size and shape of the reconstructed ventricle and avoid both undercorrection and excessive reduction in the residual chamber [16,17]. In our case, this step was particularly important because the aneurysm occupied a very large proportion of the left ventricular cavity. The balloon acted as an internal template during tightening of the circumferential 3-0 Prolene purse-string suture placed at the transition zone between fibrotic aneurysmal tissue and viable myocardium. This allowed controlled exclusion of the non-contractile aneurysmal segment, restoration of a more physiological ellipsoidal ventricular geometry, preservation of an adequate functional residual cavity, and maintenance of the long-axis orientation of the left ventricle. After completion of the reconstruction, the residual aneurysmal wall was sutured over the repair, providing an additional external haemostatic and reinforcing layer, which was particularly relevant given the thin fibrotic wall and the presence of partial thrombosis within the aneurysmal sac [18,19,20,21,22].

The present case illustrates that even patients presenting with extreme ventricular dilatation (LVEDV > 1100 mL), severely depressed LVEF (12%), multiorgan dysfunction, and imaging features suggestive of contained free-wall rupture may still achieve substantial functional recovery when meticulous patient selection, multimodality imaging, complete revascularization, and individualized ventricular reconstruction are combined. This favorable outcome highlights the importance of anatomical rather than solely functional assessment when considering surgical ventricular reconstruction.

The role of surgical ventricular reconstruction remains controversial after the STICH trial, which demonstrated no additional survival benefit of adding SVR to CABG in an unselected population with ischemic cardiomyopathy. However, many patients enrolled in STICH did not present with giant true ventricular aneurysms such as the present case. Therefore, carefully selected patients with large dyskinetic aneurysms, severe ventricular distortion, intraventricular thrombus, or mechanical complications may still benefit from surgical reconstruction when performed in experienced centers [4,23,24,25].

The postoperative course further highlights the importance of anticipating temporary circulatory support after surgical ventricular reconstruction in high-risk patients. Despite effective restoration of left ventricular geometry and an immediate improvement in left ventricular systolic function, the patient required inotropic and vasopressor support, inhaled nitric oxide for right ventricular dysfunction and pulmonary hypertension, and intra-aortic balloon pump support during the early postoperative period. This need for temporary support was consistent with the severity of preoperative biventricular dysfunction and systemic hypoperfusion. However, rapid weaning of vasoactive medication by the second postoperative day and removal of the intra-aortic balloon pump on the third postoperative day suggested favourable haemodynamic adaptation after reconstruction.

At the six-month follow-up, the patient remained free of overt heart failure symptoms, with improvement of left ventricular ejection fraction to 45%. This outcome supports the concept that, in carefully selected patients with a clearly demarcated scarred aneurysmal segment and residual viable myocardium, surgical ventricular reconstruction may provide meaningful functional recovery even in the setting of severely depressed preoperative ejection fraction. Nevertheless, appropriate patient selection, detailed multimodality imaging, meticulous surgical planning, and postoperative optimisation of guideline-directed medical therapy remain essential determinants of outcome.

This report has several limitations. First, it describes the experience of a single patient treated at a single tertiary referral center without a matched comparator group; therefore, the findings cannot be generalized to all patients with post-infarction left ventricular aneurysms. Second, because surgical ventricular reconstruction was performed in combination with left internal thoracic artery bypass grafting to the left anterior descending artery, the individual contribution of balloon-guided ventricular reconstruction to the observed haemodynamic and functional improvement cannot be determined. Third, postoperative follow-up was limited to six months, as longer-term clinical and imaging data were unavailable at the time of manuscript preparation. Consequently, long-term outcomes, including recurrent ventricular arrhythmias, thrombus recurrence, heart failure rehospitalization, and sustained ventricular function beyond six months, could not be assessed. Furthermore, postoperative cardiac magnetic resonance imaging was not performed; therefore, it was not possible to determine the relative contribution of geometric ventricular restoration versus recovery of hibernating myocardium after surgical revascularization to the observed improvement in left ventricular ejection fraction. Finally, although balloon-guided endoventricular reconstruction facilitates restoration of ventricular geometry and controlled sizing of the residual cavity, the technique may be associated with increased procedural complexity, potentially resulting in longer operative and cardiopulmonary bypass times and a higher risk of intraoperative bleeding in selected patients.

## 4. Conclusions

Giant post-infarction left ventricular aneurysm remains a rare but life-threatening complication of delayed myocardial infarction, capable of producing severe ventricular distortion, refractory heart failure, thrombotic complications, and secondary right ventricular dysfunction. This case demonstrates that, in carefully selected patients with a clearly demarcated aneurysmal scar and residual viable myocardium, surgical ventricular reconstruction may represent an effective therapeutic option even in the setting of severely depressed preoperative left ventricular function. Balloon-guided endoventricular reconstruction allows controlled restoration of ventricular geometry, avoidance of excessive cavity reduction, and preservation of an adequate functional residual ventricle. Although the role of surgical ventricular reconstruction remains debated after the STICH trial, selected patients with large dyskinetic or aneurysmal segments, mechanical complications, and advanced heart failure may still benefit from an individualized surgical approach. Multimodality imaging, meticulous surgical planning, appropriate intraoperative sizing, complete myocardial revascularization when feasible, and postoperative guideline-directed medical therapy are essential to optimize outcomes.

## Data Availability

The raw data supporting the conclusions of this article will be made available by the authors on request.
